# Comparison of QT peak and QT end interval responses to autonomic adaptation in asymptomatic LQT1 mutation carriers

**DOI:** 10.1111/j.1475-097X.2010.01002.x

**Published:** 2011-05

**Authors:** Petri Haapalahti, Matti Viitasalo, Merja Perhonen, Heikki Väänänen, Markku Mäkijärvi, Heikki Swan, Lauri Toivonen

**Affiliations:** 1 Department of Cardiology, Helsinki University HospitalHelsinki; 2 Laboratory of Clinical Physiology, Helsinki University HospitalHelsinki; 3 Laboratory of Biomedical Engineering, Helsinki University of TechnologyEspoo; 4 BioMag Laboratory, Helsinki University HospitalHelsinki, Finland

**Keywords:** autonomic nervous system, electrocardiography, long QT Syndrome, QT interval

## Abstract

**Background:**

LQT1 subtype of long QT syndrome is characterized by defective I_Ks_, which is intrinsically stronger in the epicardium than in the midmyocardial region. Electrocardiographic QT peak and QT end intervals may reflect complete repolarization of epicardium and midmyocardial region of the ventricular wall, respectively. Repolarization abnormalities in LQT1 carriers may therefore be more easily detected in the QT peak intervals.

**Methods:**

Asymptomatic KCNQ1 mutation carriers (LQT1, *n* = 9) and unaffected healthy controls (*n* = 8) were studied during Valsalva manoeuvre, mental stress, handgrip and supine exercise. Global QT peak and QT end intervals derived from 25 simultaneous electrocardiographic leads were measured beat to beat with an automated method.

**Results:**

In unaffected subjects, the percentage shortening of QT peak was greater than that of QT end during mental stress and during the recovery phases of Valsalva and supine exercise. In LQT1 carriers, the percentage shortening of the intervals was similar. At the beginning of Valsalva strain under abrupt endogenous sympathetic activation, QT peak shortened in LQT1 but not in control patients yielding increased electrocardiographic transmural dispersion of repolarization in LQT1.

**Conclusions:**

In asymptomatic KCNQ1 mutation carriers, repolarization abnormalities are more evident in the QT peak than in the QT end interval during adrenergic adaptation, possibly related to transmural differences in the degree of I_Ks_ block.

## Introduction

Effects of the autonomic nervous system on LQT1 physiology are of specific interest, because in patients with LQT1 subtype of long QT syndrome, syncope or sudden death often occurs during exercise or psychological stress (Schwartz *et al.*, 2001). Accordingly, in particular, sympathetic stimulation unmasks the abnormal repolarization in LQT1 (Shimizu & Antzelevitch, 2000; Tanabe *et al.*, 2001). We have recently described the effects of a series of standardized non-invasive cardiovascular autonomic function tests on the QT intervals of asymptomatic LQT1 mutation carriers, using beat-to-beat measurements (Haapalahti *et al.*, 2006). We observed impaired QT shortening during and exaggerated QT prolongation after autonomic manoeuvres in LQT1 carriers. Observations from these experiments also suggested that the responses of the QT peak intervals might be more blunted than the responses of the QT end intervals. In the present extension of our previous study, we set out to test the hypothesis that the repolarization abnormality in LQT1 mutation carriers would be more readily observed in the QT peak interval.

## Methods

### Patient population

Nine asymptomatic LQT1 carriers (LQT1) with the same C-terminal mutation (G589D) (Piippo *et al.*, 2001) and eight healthy unaffected subjects (control) participated in the study. The mean age of the LQT1 group was 41 ± 12 years (range 23–52 years), whereas the mean age of the control group was 44 ± 10 years (range 24–58 years). Seven of the LQT1 carriers and six of the controls were women. All LQT1 carriers had normal or only slightly prolonged baseline QTc intervals (mean 445 ± 21 ms). Baseline QTc was 405 ± 21 ms in controls. None of the participants showed signs of ischaemia during a maximal exercise test or signs of structural heart disease in cardiac magnetic resonance imaging. None of the study subjects took any medications, and they avoided alcohol for 24 h, caffeinated beverages, or a heavy meal for 6 h before the study, as well as extreme physical activity on the study day. Written informed consent was obtained, and the study protocol was approved by the local ethics committee.

### ECG recordings and automated QT interval measurements

The autonomic function tests used for sympathetic activation, as well as the methods for signal acquisition and processing have been described in more detail earlier (Haapalahti *et al.*, 2006). Briefly, modified body surface potential recordings consisting of 25 chest leads were obtained during a Valsalva manoeuvre (40 mmHg expiration pressure for 15 s), mental stress (3 min of verbal serial subtraction), sustained handgrip (3 min at 30% of maximal grip strength) and light supine bicycle exercise (10 min at 70% of predetermined maximal exercise heart rate). All the tests were performed on the same day in the above-mentioned order, with a sufficient period of rest in between to allow for stabilization of basic physiological state. A 60-s baseline recording was obtained before each test. We triggered the signals to the steepest upward slope of the R wave and subtracted a spline-fitted baseline. To reduce noise, we used a 5-beat moving average filtering method (two preceding and two subsequent QRST complexes), except during the Valsalva manoeuvre. The intervals from the trigger point to the peak (QT peak) and the end (QT end) of the T wave were automatically measured from every heart beat on every channel, with a previously validated algorithm for the determination of T-wave fiducial points (Oikarinen *et al.*, 1998; Viitasalo *et al.*, 2002; Hekkala *et al.*, 2006). The peak of the T wave was defined as the apex of the parabola fitted to the highest amplitude change after the QRS, whereas the end of the T wave was identified to the point where the steepest tangent after the peak crossed the baseline. In case of asymmetric T waves, the second derivative of the signal was used to detect discontinuities after the peak. ECG leads with excessive noise or systematically misinterpreted time points were excluded from further analysis.

### Data processing and statistical analysis

We averaged the QT peak and QT end intervals for each heart beat over all leads to obtain a beat-to-beat time series of global QT intervals. For statistical comparisons, we averaged values over defined time periods. These defined time periods were 5 or 10 s during Valsalva, 30 s during mental stress, sustained handgrip and 60 s during recovery from exercise. Using these defined time periods, we calculated the relative changes of QT peak and QT end in per cent from baseline value (ΔQTpeak and ΔQTend). In addition, we calculated the ratio between the measured QT peak and QT end intervals (QT peak/QT end ratio).

Baseline data are presented as mean ± SD, data in the figures as mean ± SEM. For statistical comparison between study groups across time, we further calculated an individual difference score (ΔQTend-ΔQTpeak) for each time period. This difference score was then used as the dependent variable in a mixed linear model, with study group as a fixed effect, time period as both fixed and repeated effect, and percentage change in heart rate as a covariate. We investigated the study group – time period interaction to detect whether the difference score behaved differently in LQT1 carriers and controls during any portion of a test. QT peak/QT end ratio was assessed similarly. For illustrative purposes, we also performed the Wilcoxon signed rank test to detect within-group differences in the relative changes of QT peak and QT end separately (without correction for multiple comparisons) at each predefined time point. Baseline group means were compared with the unpaired *t*-test. We used the SPSS 16.0.2 program (SPSS Inc., Chicago, IL, USA) for statistical analyses and considered *P*<0.05 to be statistically significant.

## Results

Baseline QT peak (339 ± 32 versus 303 ± 18 ms, *P* = 0.007) and QT end (417 ± 32 versus 378 ± 19 ms, *P* = 0.005) intervals were longer in LQT1 carriers than in control subjects. There was no difference in resting heart rate between the study groups (62 ± 5 versus 64 ± 10 beats/min, *P* = 0.36). The maximum heart rates reached during the tests were in LQT1 carriers 88 ± 12 beats/min during Valsalva manoeuvre, 80 ± 6 during mental stress, 75 ± 7 during sustained handgrip and 112 ± 7 during supine exercise. In controls, the corresponding maximum heart rates were 97 ± 13 (*P* = 0.16 compared with LQT1), 89 ± 7 (*P* = 0.01), 81 ± 12 (*P* = 0.12) and 126 ± 5 beats/min (*P* = 0.01), respectively.

**Figure 1:**
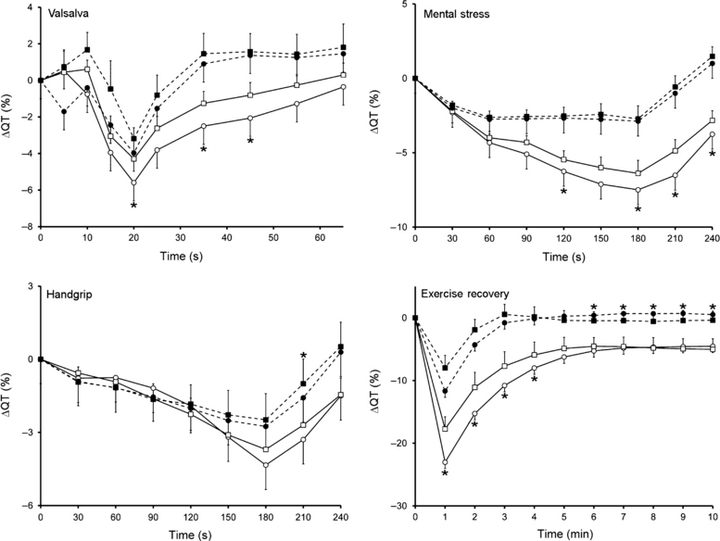
Percentage change of QT peak (circles) and QT end (squares) intervals during Valsalva manoeuvre, mental stress, handgrip and exercise recovery in LQT1 carriers (closed symbols) and controls (open symbols). Data points represent averages of 5 or 10 s during Valsalva, 30 s during mental stress and handgrip, and 60 s during exercise recovery. *P<0.05 ΔQTend versus ΔQTpeak.

Figure 1 compares ΔQTpeak and ΔQTend during the interventions. Excessive noise prevented reliable QT measurements during supine exercise; therefore, we analysed only the recovery phase. There was a statistically significant difference in the overall behaviour of QT end-QT peak difference score (not shown) between LQT1 and controls during mental stress (*P* = 0.009) and recovery from exercise (*P* = 0.001). Control subjects exhibited a greater relative shortening (in percentage) of QT peak than of QT end intervals during these tests. However, this response pattern was absent in LQT1 carriers, who showed similar percentage shortening of QT peak and QT end and even less shortening of QT peak than QT end during late recovery from exercise. During sustained handgrip, the relative changes in QT peak were similar to those of QT end in both groups. At the beginning of the Valsalva strain, LQT1 carriers but not control subjects showed shortening of the QT peak.

**Figure 2:**
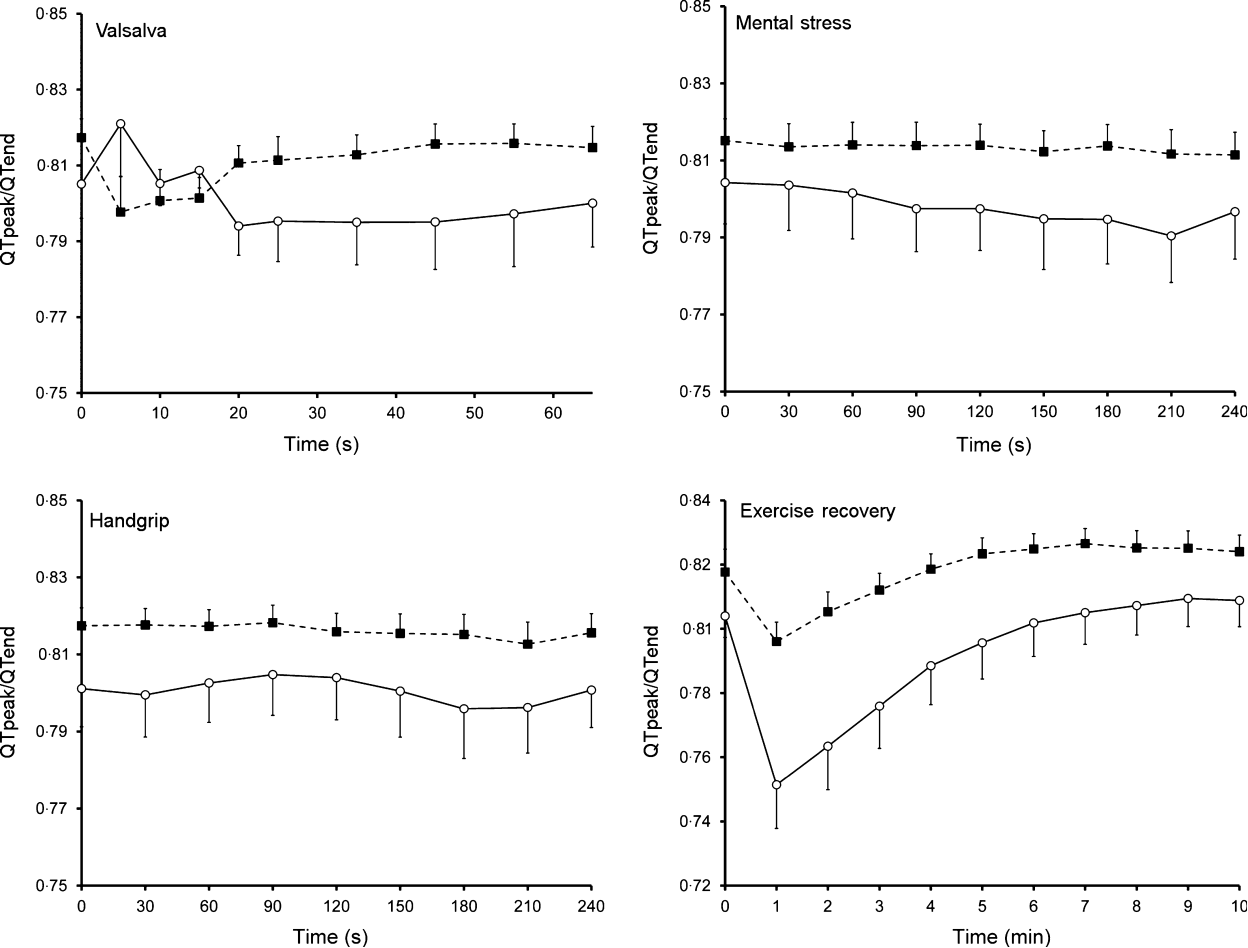
QT peak/QT end ratio during Valsalva, mental stress, handgrip and exercise recovery in LQT1 carriers (closed symbols) and controls (open symbols). *P*<0.01 for overall difference in study group*time interaction during all tests.

Figure 2 describes the behaviour of QT peak/QT end ratios during the tests. In control subjects, the QT peak/QT end ratio decreased after release of Valsalva strain, during mental stress and during recovery from supine exercise. In LQT1 carriers, however, QT peak/QT end decreased only during the strain phase of the Valsalva manoeuvre, as well as during the initial phase of recovery after exercise.

## Discussion

### Main findings

Our results show that in asymptomatic LQT1 mutation carriers, repolarization abnormalities are more easily detected in the QT peak than in the QT end interval during sympathetic activation. In control subjects, the shortening of QT peak was significantly greater than that of QT end, when viewed as a relative change compared with baseline. In LQT1 carriers, however, both QT peak and QT end intervals shortened to a similar degree, indicating impaired QT peak shortening compared to QT end.

### Normal autonomic responses of QT peak and QT end intervals

We observed a greater relative shortening of QT peak than that of QT end during mental stress and during the recovery phases of Valsalva and supine exercise in our healthy control subjects. Previous clinical experiments addressing these assumptions are scarce, however, as most previous studies have reported only rate-corrected QT intervals without direct comparison of QT peak to QT end. Sundqvist and Sylvén observed in healthy patients that QT peak and QT end intervals shortened in parallel during maximal exercise, while the QT peak/QT end ratio decreased slightly (Sundqvist & Sylvén, 1989), translating into a greater relative shortening of QT peak than QT end intervals.

In experimental studies, rate-dependent changes in epicardial action potential duration (APD) + activation time are closely approximated by the changes in the QT peak interval, whereas changes in the M cell APD + activation time are closely approximated by changes in the QT end interval (Yan & Antzelevitch, 1998). In addition, APD rate dependence is steeper in M cells than in epicardial cells (Yan & Antzelevitch, 1998), whereas ß-adrenergic stimulation with isoprenaline at constant cycle length homogenously abbreviates the APD in both cell types (Shimizu & Antzelevitch, 1998). Extrapolating these observations to ECG, an increase in heart rate alone would then be expected to abbreviate QT end more than QT peak, whereas both intervals would be expected to shorten similarly in response to ß-adrenergic stimulation under constant heart rate. Thus, our findings are in strong accord with previous experimental observations. It should be emphasized here that a given absolute shortening (in milliseconds) of both the shorter QT peak interval and the longer QT end interval corresponds to a markedly greater relative shortening (in percentage) of the former.

### Autonomic QT peak and QT end responses in LQT1 carriers

In the present extension of our previous study showing impaired shortening of QT intervals to autonomic manoeuvres in asymptomatic LQT1 carriers, we further observed that LQT1 carriers exhibit similar percentage shortening of QT peak and QT end intervals during mental stress and during the recovery from Valsalva instead of a greater percentage shortening of QT peak than QT end as was observed in the control patients during these tests. These findings indicate that in LQT1 carriers, QT peak responses are even more impaired than QT end responses. In addition, during the initial strain phase of Valsalva with abrupt endogenous sympathetic activation, we observed a significant decrease in QT peak/QT end ratio resulting in prolongation of the T-wave peak to T-wave end interval and thus an increased transmural dispersion of repolarization in LQT1 carriers. In control patients, the behaviour of the QT peak/QT end ratio was reversed at the beginning of Valsalva strain (Fig. 2).

LQT1 is characterized by defective slowly activating delayed rectifier current (I_Ks_) (Wang *et al.*, 1996), which is intrinsically stronger in the epicardium than in the midmyocardial region (Liu & Antzelevitch, 1995). In an experimental LQT1 model, inhibition of I_Ks_ prolongs the APD homogenously in epicardial, endocardial and M cells at a wide range of cycle lengths, without increasing transmural dispersion of repolarization (Shimizu & Antzelevitch, 1998). The inhibition of I_Ks_ thereby causes a greater relative prolongation of the shorter epicardial APD than of the longer M-cell APD. Extrapolating this to baseline clinical ECG, LQT1 carriers show both prolonged QT peak and prolonged QT end intervals without a change in T-wave peak to T-wave end interval. During strong sympathetic stimulation, however, direct stimulation of ß-adrenergic receptors with isoprenaline abbreviates the APD of epicardial and endocardial cells, but not the APD of M cells (Shimizu & Antzelevitch, 1998). This is thought to arise from a larger augmentation of residual I_Ks_ in epicardial than in M cells, offsetting the balance of ionic currents during repolarization in the latter. Concordantly, in clinical studies among LQT1 carriers, both adrenaline infusion (Tanabe *et al.*, 2001) and exercise stress (Takenaka *et al.*, 2003) prolonged the T-wave peak to T-wave end interval, apparently owing to shortening of QT peak but not of QT end. The present finding of the abrupt QT peak shortening during the initial strain of Valsalva is in accordance with these previous experimental and clinical observations. Thus, our results suggest that the abnormal repolarization in LQT1 carriers is more readily manifested in the QT peak than in the QT end interval. In view of the fact that it was apparent in asymptomatic carriers with nearly normal baseline QT intervals, this characteristic could be useful in the evaluation of suspected LQT1 mutation carriers.

As we have reported earlier (Haapalahti *et al.*, 2006), heart rate acceleration during sympathetic activation was attenuated in LQT1 carriers compared to controls, probably owing to decreased I_Ks_ in the sinus node. Although a potential confounder, this between-groups difference should not invalidate the comparison of relative changes of QT peak with QT end within a group. Moreover, we observed earlier (Haapalahti *et al.*, 2006) that in control subjects the QT peak/HR slopes were no different from the QT end/HR slopes, suggesting that any differences observed in QT peak/QT end ratios are most likely dependent on other factors than heart rate. As a result of the complex relation between QT intervals and heart rate during autonomic adaptation (Davidowski & Wolf, 1984), we assessed only uncorrected QT intervals. In fact, the diverging dynamics of QT peak and QT end observed in our LQT1 carriers strongly argues against the use of current heart rate correction formulas on QT peak and especially T-wave peak to T-wave end interval measurements.

### Limitations of the study

We studied only a limited number of asymptomatic carriers of a single KCNQ1 mutation, so the present results might not be applicable to all subjects with the LQT1 genotype. However, the homogenous study population limits confounding genotype-specific variability on ventricular repolarization. A larger number of patients with different mutations would be needed to assess the usefulness of the present approach in the diagnosis and management of LQT1.

Our interpretation of the results relies on the assumption that QT peak and QT end on the surface ECG represent repolarization of epicardium and M region, respectively. However, this model is based on the so-called left ventricle wedge preparation of a part of the canine left ventricular wall. During recent years, the relevance of this model in the intact body has been challenged. By using *in vivo* pig models, Xia *et al.*, (2005a,b); observed that the T-wave peak coincides with the earliest end of repolarization in intact pig heart but not with full repolarization of the epicardium. Opthof *et al.* (2007) reported that parts of the intact canine heart fully repolarize before the moment of the T-wave peak. Nevertheless, our simplification of the electrophysiology of the T-wave peak does not invalidate our main finding that in asymptomatic LQT1 mutation carriers, repolarization abnormalities are more easily detected in the QT peak than in the QT end interval during sympathetic activation.
